# KRAS Genotype Correlates with Proteasome Inhibitor Ixazomib Activity in Preclinical *In Vivo* Models of Colon and Non-Small Cell Lung Cancer: Potential Role of Tumor Metabolism

**DOI:** 10.1371/journal.pone.0144825

**Published:** 2015-12-28

**Authors:** Nibedita Chattopadhyay, Allison J. Berger, Erik Koenig, Bret Bannerman, James Garnsey, Hugues Bernard, Paul Hales, Angel Maldonado Lopez, Yu Yang, Jill Donelan, Kristen Jordan, Stephen Tirrell, Bradley Stringer, Cindy Xia, Greg Hather, Katherine Galvin, Mark Manfredi, Nelson Rhodes, Ben Amidon

**Affiliations:** 1 Takeda Pharmaceuticals International Co., Cambridge, Massachusetts, United States of America; 2 Metabolon Inc, Durham, North Carolina, United States of America; University of Florida, UNITED STATES

## Abstract

In non-clinical studies, the proteasome inhibitor ixazomib inhibits cell growth in a broad panel of solid tumor cell lines *in vitro*. In contrast, antitumor activity in xenograft tumors is model-dependent, with some solid tumors showing no response to ixazomib. In this study we examined factors responsible for ixazomib sensitivity or resistance using mouse xenograft models. A survey of 14 non-small cell lung cancer (NSCLC) and 6 colon xenografts showed a striking relationship between ixazomib activity and *KRAS* genotype; tumors with wild-type (WT) *KRAS* were more sensitive to ixazomib than tumors harboring *KRAS* activating mutations. To confirm the association between *KRAS* genotype and ixazomib sensitivity, we used SW48 isogenic colon cancer cell lines. Either KRAS-G13D or KRAS-G12V mutations were introduced into KRAS-WT SW48 cells to generate cells that stably express activated KRAS. SW48 KRAS WT tumors, but neither SW48-KRAS-G13D tumors nor SW48-KRAS-G12V tumors, were sensitive to ixazomib *in vivo*. Since activated KRAS is known to be associated with metabolic reprogramming, we compared metabolite profiling of SW48-WT and SW48-KRAS-G13D tumors treated with or without ixazomib. Prior to treatment there were significant metabolic differences between SW48 WT and SW48-KRAS-G13D tumors, reflecting higher oxidative stress and glucose utilization in the KRAS-G13D tumors. Ixazomib treatment resulted in significant metabolic regulation, and some of these changes were specific to KRAS WT tumors. Depletion of free amino acid pools and activation of GCN2-eIF2α-pathways were observed both in tumor types. However, changes in lipid beta oxidation were observed in only the KRAS WT tumors. The non-clinical data presented here show a correlation between *KRAS* genotype and ixazomib sensitivity in NSCLC and colon xenografts and provide new evidence of regulation of key metabolic pathways by proteasome inhibition.

## Introduction

The ubiquitin proteasome system processes the majority of cellular proteins, including proteins involved in growth, cell cycle regulation and apoptosis [1,2,3]. VELCADE (bortezomib) is the first-in-class proteasome inhibitor (PI), approved for the treatment of patients with multiple myeloma (MM) [4] and mantle cell lymphoma [1,5,6]. Ixazomib is an investigational oral PI, currently in phase III clinical trials in patients with MM and light chain amyloidosis. PIs have demonstrated a wide range of effects on MM cells and their bone marrow microenvironment. This includes effects on cell cycle, NF-kB inhibition, and apoptotic regulators as well as induction of the integrated stress response, inhibition of IL-6 production and signaling, and many others [7,8]. As highly secretory antibody-producing cells, MM cells have an elevated need for protein quality control and may have a greater reliance on proteasome function for survival compared to other cell types [9].

Although PI’s have been tested in various solid tumor clinical trials, no PI is currently approved for treatment of solid tumors. Preclinical activity of ixazomib and other PIs has been demonstrated in a limited number of solid tumor xenograft models [10,11,12], but preclinical identification of genetic and phenotypic determinants of solid tumor sensitivity to PIs has been lacking.

In this study we report a striking correlation between *KRAS* genotype and ixazomib sensitivity in a panel of non small cell lung cancer (NSCLC) and colon xenograft models. Ixazomib showed significantly better antitumor activity in wild-type (WT) KRAS xenografts than in xenografts with activating KRAS mutations. Notably, this association was not observed *in vitro*. Approximately 25–30% of human tumors are estimated to harbor activating RAS mutations [13]. Of the three RAS isoforms (KRAS, NRAS and HRAS), *KRAS* is most frequently mutated in malignancies. *KRAS* mutation is especially predominant in colon (40–45%), NSCLC (16–40%) and pancreatic ductal carcinoma (69–95%) [14]. The RAS proteins are GTPases which regulate a number of cellular processes including proliferation, survival, growth, migration, differentiation and metabolism. Mutations in the *KRAS* oncogene are known to modulate a number of major metabolic pathways, including glycolysis, tricarboxylic acid (TCA) cycle, pentose phosphate pathway (PPP), membrane biogenesis as well as glucose transport [15,16,17]. To characterize the mechanism of KRAS-associated *in vivo* resistance to ixazomib, we compared the metabolic profile at baseline and following ixazomib treatment in isogenic KRAS WT and mutant SW48 xenografts. We identified several key metabolic pathways that are differentially impacted by KRAS status, ixazomib treatment, or both. The data suggest that these metabolic pathways might play a role in determining sensitivity to proteasome inhibitor in solid tumors.

## Methods and Materials

### Cell lines and reagents

SW48 and SW48-KRAS-G13D and SW48-KRAS-G12V cells were obtained from Horizon Discovery Ltd. Cambridge, UK and maintained in McCoy’s 5A media supplemented with 10% serum.

The 8 other cell lines used as xenografts were obtained from ATCC, Manassas, VA.

For *in vitro* use, ixazomib was formulated in DMSO and diluted in media to desired concentration.

### Antibodies

Rabbit antibodies against human GLUT1, GLUT 4, GCN2, pGCN2 (T899) and eIF2α were obtained from Abcam, Cambridge, MA. FASN, pACC (S79), ACC, CPT-1 antibodies were obtained from Cell Signaling Technology, Danvers, MA and mouse antibody against human peIF2α (S52) was obtained from Invitrogen, Grand Island, NY. All the antibodies were used at 1:1000 dilution.

### In vivo studies in xenograft bearing mice

All the animal research and veterinary care that were performed at Takeda Boston, was conducted under an approved Takeda Boston Institutional Animal Care and Use committee (IACUC) protocol in an Association for Assessment and Accreditation of Laboratory animal Care International (AAALAC) accredited facility. Immunocompromised mice were housed in a controlled environment and received food and water ad libitum. The details of the xenograft models including indication, number of cells implanted and host strain are specified in S1 Table.

Animal studies that were performed at Oncotest, GmBH was conducted according to the guidelines of German Animal Welfare Act (Tierschutzgesetz).

For cell line xenografts, a defined number of cells (with or without Matrigel, BD Biosciences, Bedford, MA) were subcutaneously inoculated into the right flank of mice and tumor growth was monitored with caliper measurement. Once the mean tumor reached a certain volume (between 120-250mm^3^, depending on the model), animals were randomized into different treatment groups of 8–10 animals per group. Primary human tumor xenograft models annotated with PHTX were developed at Takeda. The patient samples were obtained from either the National Disease Research Interchange, Philadelphia, PA or the Cooperative Human Tissue Network, NCI. Surgically removed patient samples were subcutaneously implanted in mice and passaged several times (no more than 10) before using in efficacy studies. Primary tumors annotated with LX were developed and tested at Oncotest GmBH, Germany.

Ixazomib for *in vivo* use was formulated in 10% HP-β-CD (hydroxypropyl beta cyclodextrin) and dosed at its maximum tolerated dose (MTD) for the specified mouse strain and model (between 11 to 14mg/kg, IV, BIW for 3–4 weeks).

Antitumor activity was determined by calculating the treatment over control (T/C) tumor volume ratio at the end of the study.

Either at the end of each study, of if any animal reached a humane endpoint, animals were euthanized with CO_2_ followed by cervical dislocation or another secondary method of euthanasia approved by the IACUC protocol.

### Cell viability assay

Cells were grown in their respective growth media, supplemented with 10% fetal bovine serum and seeded on 384-well poly-D-lysine (PDL)-coated black, clear-bottom plates (BD BioCoat^™^) and incubated 24 h at 37°C, 6% CO_2_. Cells were then treated with ixazomib at various concentrations for 72 hrs. Viability was assessed with CellTiter-Glo^®^ Cell Viability reagent according to manufacturer’s instructions (Promega, Madison, WI).

### 2D Colony formation assay

5000 cells per well of wild type or KRAS mutant (G12V or G13D) SW48 lines were seeded in 12-well plates (BD Falcon, BD Biosciences, San Jose, CA) and incubated overnight. Cell were then treated with 0–500 nM of ixazomib for 11 days and fixed in 4% PFA before staining with 0.5% crystal violet in 25% methanol. Area of the colonies was measured using Metamorph software and IC_50_ was determined by calculating the concentration at which the percentage average colony area reached 50% relative to vehicle control.

3D colony formation assay for 20 primary patient derived NSCLC tumors was described in [18].

### Analysis of tumor pharmacokinetics (PK) and 20S proteasome activity

Tumor PK and 20S assays are previously described [10]. Briefly, tumor samples were harvested at different time points after vehicle or drug administration and homogenized in mouse plasma for PK anaylsis. Concentration of ixazomib in the tumors was determined using LC/MS/MS methods.

20S proteasome β5 enzymatic activity was measured after homogenizing tumor samples in buffer containing HEPES and DTT using a Covaris sonicator.

### Mutational analysis

DNA from xenograft tumors was amplified and analyzed by Sequenom assay as described in [19]. The gene panel included in the OncoCarta^™^ V1 and custom panel is provided in S2 Table. For the *KRAS* gene, the mutations tested are G12 A/C/D/F/R/S/V, G13 A/C/D/R/S/V, L19F, Q22K, T58I, A59T/V, G60D, Q61 E/H/K/L/P/R and A146T.

### Metabolic profiling

Tumor samples were harvested at different time points after IV administration of a single dose of ixazomib. For vehicle control, two time points (1hr and 8hrs) were evaluated. The tumor lysis and global metabolic profiling analysis were done at Metabolon Inc. In brief, samples were extracted in methanol and divided into equal parts for analysis on the GC/MS and LC/MS/MS platforms. Proprietary software was used to match ions to an in-house library of standards for metabolite identification and for metabolite quantitation by peak area. Welch’s two sample t-tests and two-way ANOVA were used to compare the means of two populations. p values ≤0.05 were considered highly significant and p values between 0.05 and 0.1 were considered less significant.

### Western blot analysis

Tumor samples were homogenized in MPER buffer containing protease inhibitors [10]. 10–20 μgm of proteins were loaded onto 4% to 12% Bis-Tris gels, or 3–8% tris acetate gels for higher molecular weight proteins (Invitrogen). Proteins were transferred to PVDF-FL membranes (Millipore), blocked and incubated with primary antibodies followed by Alexa Fluor 680 labelled secondary antibody (Molecular Probes). Bands were detected using Odyssey infrared imaging system (LI-COR Biosciences, Lincon, NE).

## Results

### KRAS mutations correlate with decreased sensitivity to ixazomib

**Fig 1:**
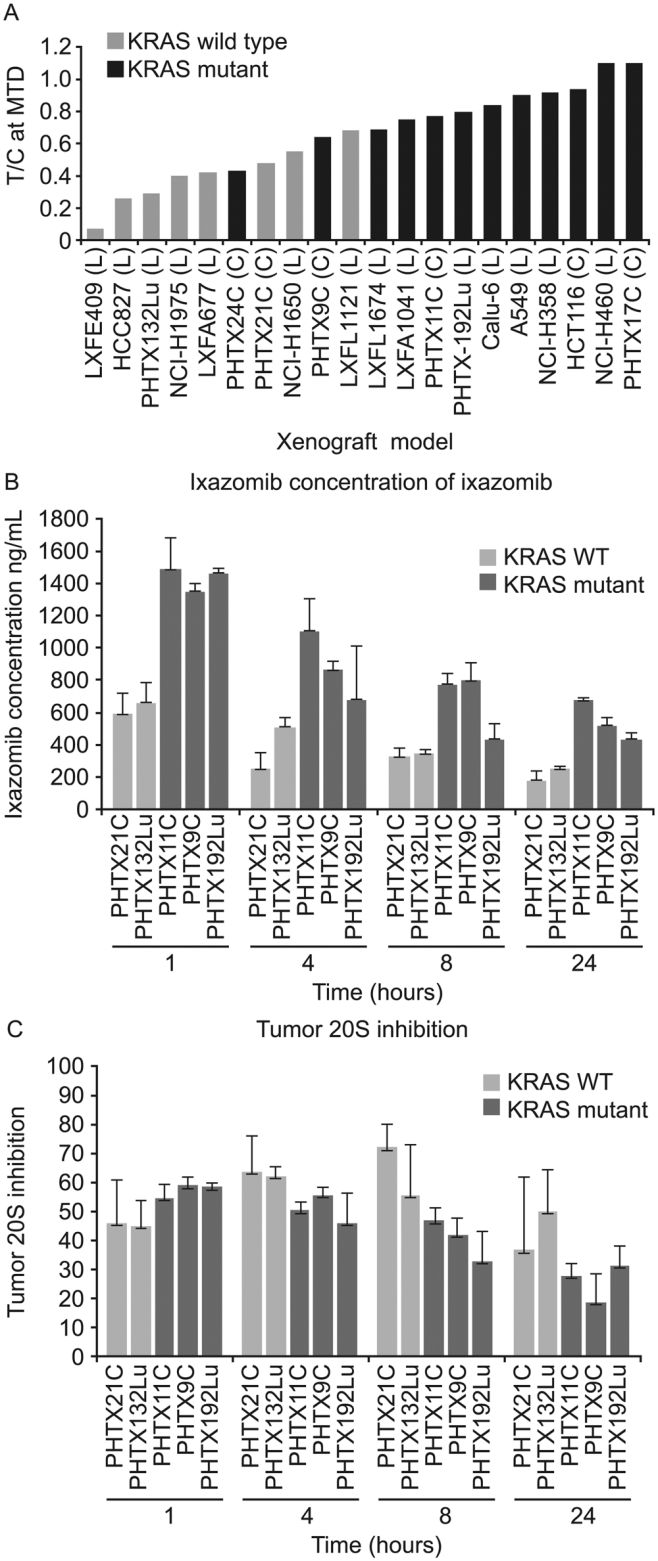
Ixazomib activity in tumor xenografts. (A) Antitumor activity of ixazomib in 14 non small cell lung (NSCLC) and 6 colon xenograft models. The panel included both cell line-derived and primary human xenografts harboring either WT or mutant KRAS. Tumor-bearing animals (n = 8–10 per group) were treated with either vehicle or MTD dose of ixazomib (between 11 and 14 mg/kg, IV, BIW) for approximately three weeks. T/C was calculated by dividing average tumor volume of drug treated animals by that of vehicle treated animals on day 19–21. Tumor concentration of ixazomib (B) and 20S proteasome inhibition (β5 site) (C) in various NSCLC and colon xenografts at different time points following a single IV administration of MTD dose of ixazomib. % 20S β5-inhibition was calculated by considering the average (n = 3 for treatment group or 4 for vehicle group) tumor 20S activity of vehicle treated animals as 100%. Each bar represents the average concentration of ixazomib or 20S inhibition in the tumor from 3–4 different animals +/- SD.

To understand the molecular factors determining sensitivity to ixazomib *in vivo*, we surveyed antitumor activity in a panel of colon and NSCLC xenograft models including primary human xenografts and cell line-derived xenografts. The antitumor activity of the drug was expressed as T/C ratio; with T/C of 0 indicating complete elimination of tumor, and T/C of 1 indicating no effect. (Fig 1A)

Mutations in a panel of oncogenes and tumor suppressors (S2 Table) were examined in the tumor xenografts by Sequenom mass spectrometry-based analysis. Consistent with the high prevalence of *KRAS* mutations in human colon and NSCLC tumors, 12 of the 20 models evaluated had *KRAS* mutations in codons G12, G13, G61 or A146T, whereas no *NRAS* or *HRAS* mutations were detected. Fewer samples had mutations in *PI3KCA*, or other known oncogenes (S3 Table). KRAS mutant tumors showed lower sensitivity to ixazomib (av. T/C = 0.82) compared to KRAS WT tumors (av. T/C = 0.4) (p = 0.002 determined by t-test) (Fig 1A).

We evaluated several possible explanations for differences in ixazomib sensitivity among the xenograft tumors, starting with pharmacokinetic (PK) and pharmacodynamic (PD) effects of ixazomib *in vivo* after a single MTD dose in 5 xenograft models. Ixazomib concentrations in KRAS mutant tumors were either similar to or higher than those in KRAS WT tumors (Fig 1B), and tumor proteasome β5-site activity was inhibited to a similar degree in both KRAS wild type and mutant tumors (Fig 1C). Additional evidence of proteasome inhibition and its downstream consequences was generated using an IHC assay for ATF3, which is upregulated as part of the integrated stress response following proteasome inhibition [10]. ATF3 upregulation was detected in all models, regardless of their *KRAS* mutation status or sensitivity to ixazomib (S1 Fig). Thus, the insensitivity of KRAS mutant tumors to ixazomib is not explained by lower tumor exposure or less target inhibition.

Interestingly, KRAS mutations have not been identified as a predictor of PI resistance in monolayer tissue culture viability assays. To determine whether the association between KRAS status and ixazomib sensitivity could be observed using an *in vitro* three dimensional model, a panel of 20 xenograft explants including WT KRAS and mutant KRAS were tested in a soft agar colony formation assay. The explants included 17 primary human xenograft tumors and 3 cell line derived xenograft tumors, and 8 of the 20 have also been tested for ixazomib response *in vivo*. The difference in sensitivity seen *in vivo* was not detected *in vitro*. The median IC_50_ in the colony formation assay was 99 nM (range 34–272 nM) for WT KRAS models, and 73 nM (range 38–17 5nM) for KRAS mutant models (S4 Table). (p = 0.4). These data suggest that mutant KRAS provides a survival advantage *in vivo* which is not mimicked in the soft agar assay format.

### Introduction of KRAS mutation reverses in vivo ixazomib sensitivity of KRAS WT xenograft

To further explore the connection between *KRAS* genotype and ixazomib sensitivity *in vivo*, we used a set of colon cancer cell lines which differ in KRAS status but are otherwise genetically matched. Stable derivatives of the SW48 cell line were derived by introducing a KRAS-G13D or KRAS-G12V point mutation via rrAV gene editing technology. The presence of mutations and the conservation of overall genomic profile of the engineered cells were confirmed by mutation detection and SNP analysis (data not shown). Activation of KRAS signaling in KRAS-G13D and KRAS-G12V cells compared to their WT counterpart was previously confirmed.[20]

**Fig 2:**
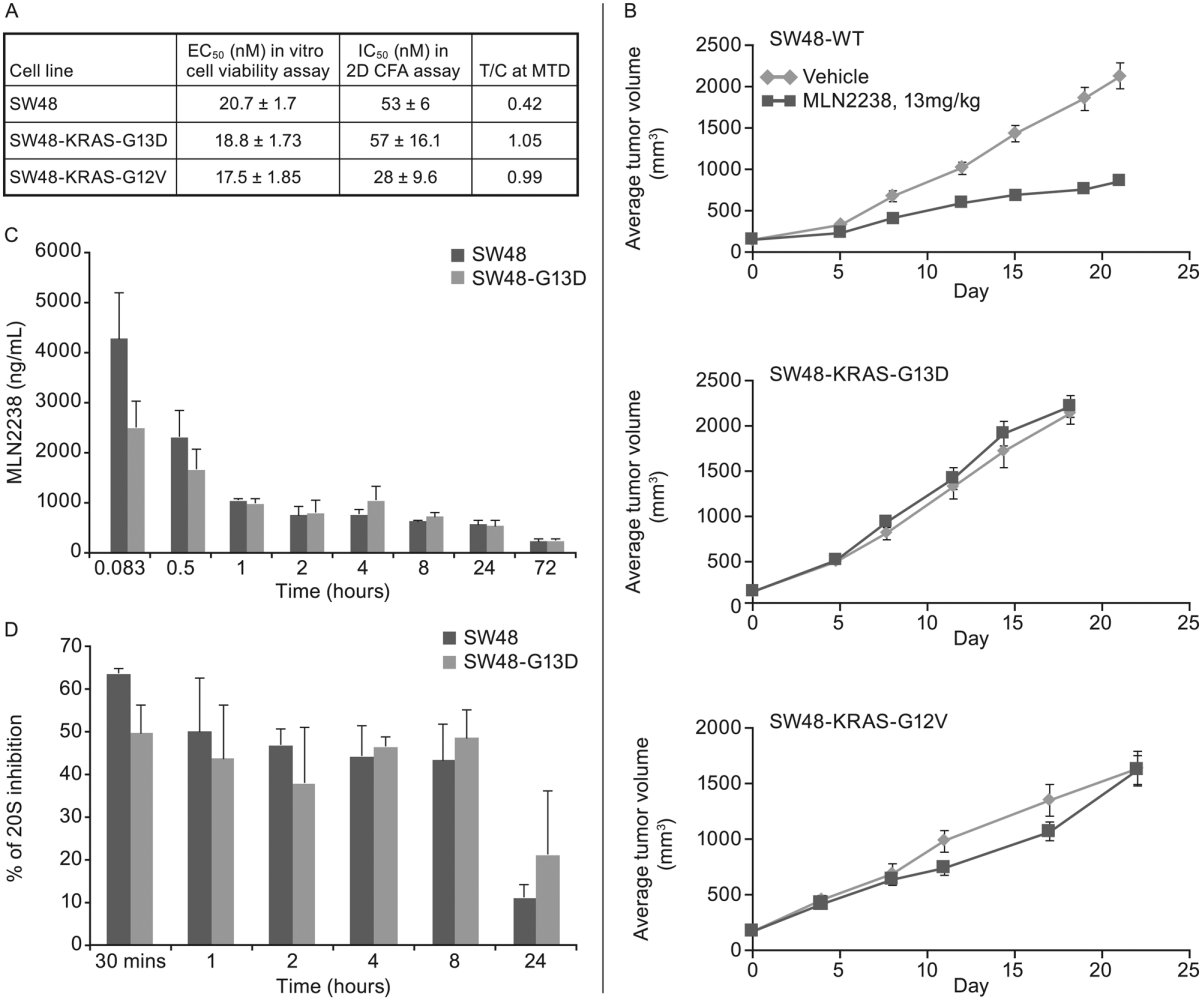
*In vitro* and *in vivo* activity of ixazomib in SW48 isogenic cells and xenografts. (A) Summary table of EC_50_, IC_50_ and T/C values for SW48, SW48-G13D and SW48-G12V cells and xenografts from *in vitro* cell viability, *in vitro* colony formation and *in vivo* antitumor activity assays. EC_50_ represents the mean concentration of ixazomib (+/-SD) required for 50% cell killing in three different experiments in cell viability assay. In the colony formation assay (CFA) the mean concentration of the drug to inhibit 50% colony formation was calculated in three different experiments and the number is mean +/-SD. 13mg/kg, IV, BIW dose was used in xenograft studies and T/C was calculated as described before. (B) Antitumor activity of ixazomib in SW48, SW48-G13D and SW48-G12V xenografts. The tumor growth over time is presented for vehicle and ixazomib treated animals bearing SW48, SW48-G13D and SW48-G12V xenografts. The treatment started when average tumor volume reached approximately 200mm^3^ and there were n = 10 animals per arm. The T/C was calculated at day 19–21. C and D: Tumor concentration of ixazomib (C) and 20S proteasome inhibition (D) at different time points following an acute IV administration of ixazomib at 13mg/kg dose in SW48 and SW48-G13D tumors. Each data point represents tumors from three different animals +/- SD

All 3 cell lines were similarly sensitive to ixazomib in the *in* vitro cell viability assay (Fig 2A). KRAS WT and KRAS-G13D cells were equally sensitive in the 2D colony formation assay, while KRAS-G12V cells were approximately 2-fold more sensitive in this CFA assay (Fig 2A). In contrast, there was a clear difference in the *in vivo* sensitivity to ixazomib in SW48, SW48-G13D and SW48-G12V xenograft tumors. SW48 xenografts with wild-type KRAS responded to ixazomib treatment with T/C of 0.42 (Fig 2B). In contrast, neither the SW48-G13D nor SW48-G12V xenograft model was sensitive to ixazomib (T/C = 1.05 and 0.99), suggesting that introduction of a *KRAS* mutation is sufficient to drive resistance to PI in tumor xenograft models *in vivo*. The 3 xenograft models showed similar growth kinetics in the vehicle groups (Fig 2B), suggesting that differences in ixazomib response are not due to differences in tumor growth rates. Furthermore, results of a PK/PD study conducted in the SW48 and SW48-G13D xenograft tumors indicate comparable levels of ixazomib exposure in the xenografts (Fig 2C), as well as similar levels of tumor proteasome inhibition (Fig 2D), consistent with results in the broader panel of xenografts described in Fig 1. Thus, the effect of the KRAS mutation is not at the level of drug exposure or target inhibition.

### Increased expression of GLUT4 receptors in KRAS mutant tumors

**Fig 3:**
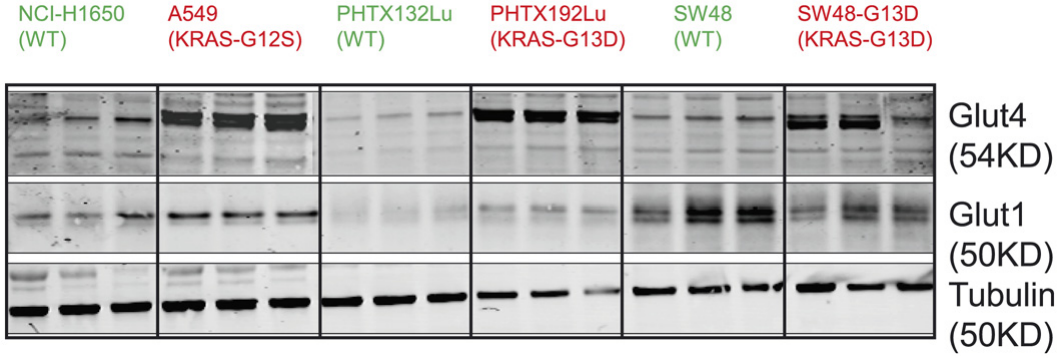
Expression of GLUT1 and GLUT4 receptors in xenograft tumors with either WT or mutant KRAS. Proteins were extracted from untreated tumors and 10μg protein was loaded in each lane. Levels of GLUT1 and GLUT4 proteins were determined by western blot and tubulin was used as a loading control. n = 3 for each tumor.

Differences in ixazomib activity *in vitro* and *in vivo* suggest factors in the tumor environment *in vivo*, such as nutrient levels, metabolites or other factors, might play a role in determining sensitivity to ixazomib. Oncogenic KRAS proteins have been reported to induce cell surface expression of glucose receptors (GLUTs), and thereby facilitate cellular glucose transport [4,15,21,22]. Therefore, we examined the baseline expression of GLUT1 and GLUT4 in a number of KRAS WT and mutant tumors by western blot (Fig 3). We included lysates from KRAS WT and mutant cell line-derived xenografts (NCI-H1650 and A549 and the isogenic pair SW48 and SW48-G13D) and primary xenografts (PHTX132Lu and PHTX192Lu). Although expression of GLUT1 receptors did not correlate with KRAS status, the KRAS mutant xenografts showed much higher levels of GLUT4 receptor expression compared to tumors with WT KRAS. Increased GLUT4 would enable increased glucose uptake, which would be advantageous in nutrient-limiting environments often found in tumors. The elevated GLUT4 expression in KRAS mutant tumors suggests that altered glucose uptake and metabolism might play some role in determining sensitivity to ixazomib.

### Global metabolic profile analysis in SW48 isogenic tumors

**Fig 4:**
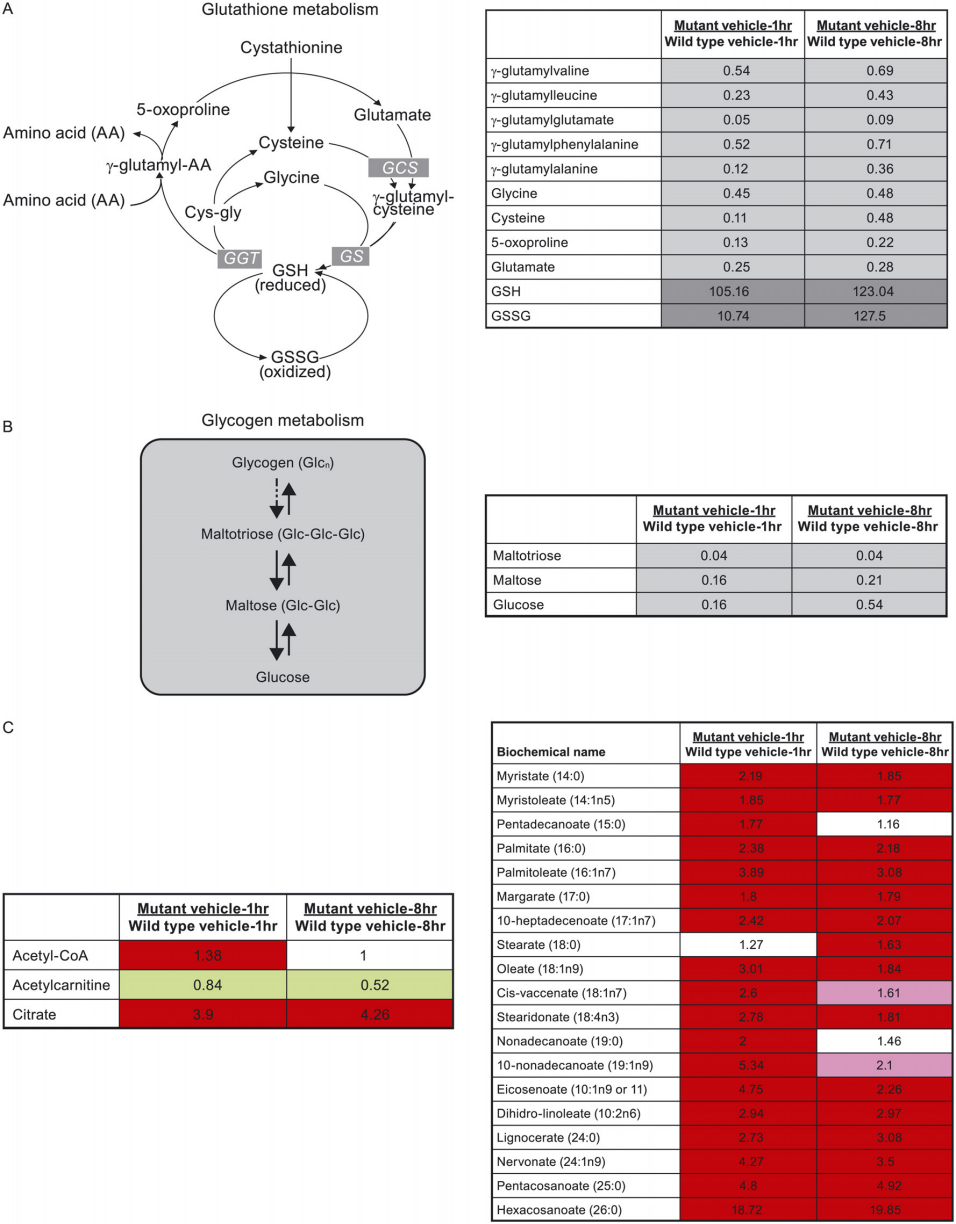
Baseline metabolic differences between SW48 KRAS WT and G13D mutant tumors. A-C: Glutathione metabolism pathways (A), glycogen metabolism (B) and relative expression of pathway metabolites in KRAS WT vs KRAS mutant tumors treated with vehicle for 1 and 8 hrs. The numbers indicate the fold change of each metabolite in KRAS mutant tumors compared to KRAS WT tumors and are the average of metabolite levels from 5 different tumors. C: Relative expression of free long chain fatty acids, acetyl coA, acetylcarnitine and citrate in KRAS mutant vs KRAS WT tumors. Green boxes indicates a ratio <1, with dark green boxes as highly significant (p ≤0.05) and light green boxes as less significant (0.05<p<0.1). Red boxes indicate a ratio >1, with dark red boxes as highly significant (p ≤0.05) and light red boxes as less significant (0.05<p<0.1). White boxes represent no change in expression.

To further understand the metabolic differences between KRAS WT and KRAS mutant tumors and their response to ixazomib, we conducted metabolic profiling on SW48 and SW48-G13D xenograft tumors harvested at different time points following a single IV dose of vehicle or ixazomib. Metabolite levels were measured by mass spectrometry-based methods and reported as a fold-change compared to defined control samples. There were a number of metabolic differences observed at baseline (vehicle-treated samples) between SW48 and SW48-G13D tumors, particularly related to glutathione and glycogen metabolism and fatty acid synthesis (Fig 4). Both oxidized and reduced glutathione (GSSG and GSH) were detected at higher levels in KRAS mutant tumors compared to KRAS WT tumors (Fig 4A). Conversely, biosynthetic precursors of glutathione, such as glutamate, cysteine, glycine, gamma-glutamyl amino acids, and 5-oxyproline were detected at lower levels in KRAS mutant xenograft tumors (Fig 4A). The elevated level of glutathione synthesis in KRAS mutant tumors may promote increased survival and drug resistance by enhancing the tumors’ ability to deal with redox stress [23].

Decreased levels of glycogen intermediates, such as maltotriose, maltose and glucose were observed in KRAS mutant tumors compared to KRAS WT tumors (Fig 4B), suggesting a rapid glycogen breakdown in KRAS mutant tumors to supply glucose. The relatively lower level of glucose present at baseline in KRAS mutant tumors is an indicator that glucose is rapidly consumed in promoting continued growth and survival.

Higher levels of free fatty acids in SW48-G13D tumors compared to SW48 (Fig 4C) were detected. Other differences in components of the fatty acid metabolism pathways were also noted; in SW48-G13D tumors, levels of citrate are higher and levels of acetylcarnitine are lower compared to the SW48 WT tumors. Taken together these differences point to increased *de novo* fatty acid synthesis in KRAS mutant tumors, which could contribute to increased membrane biosynthesis needed for continued growth and proliferation in oncogene-driven tumors.

### Amino acid depletion following ixazomib treatment

**Fig 5:**
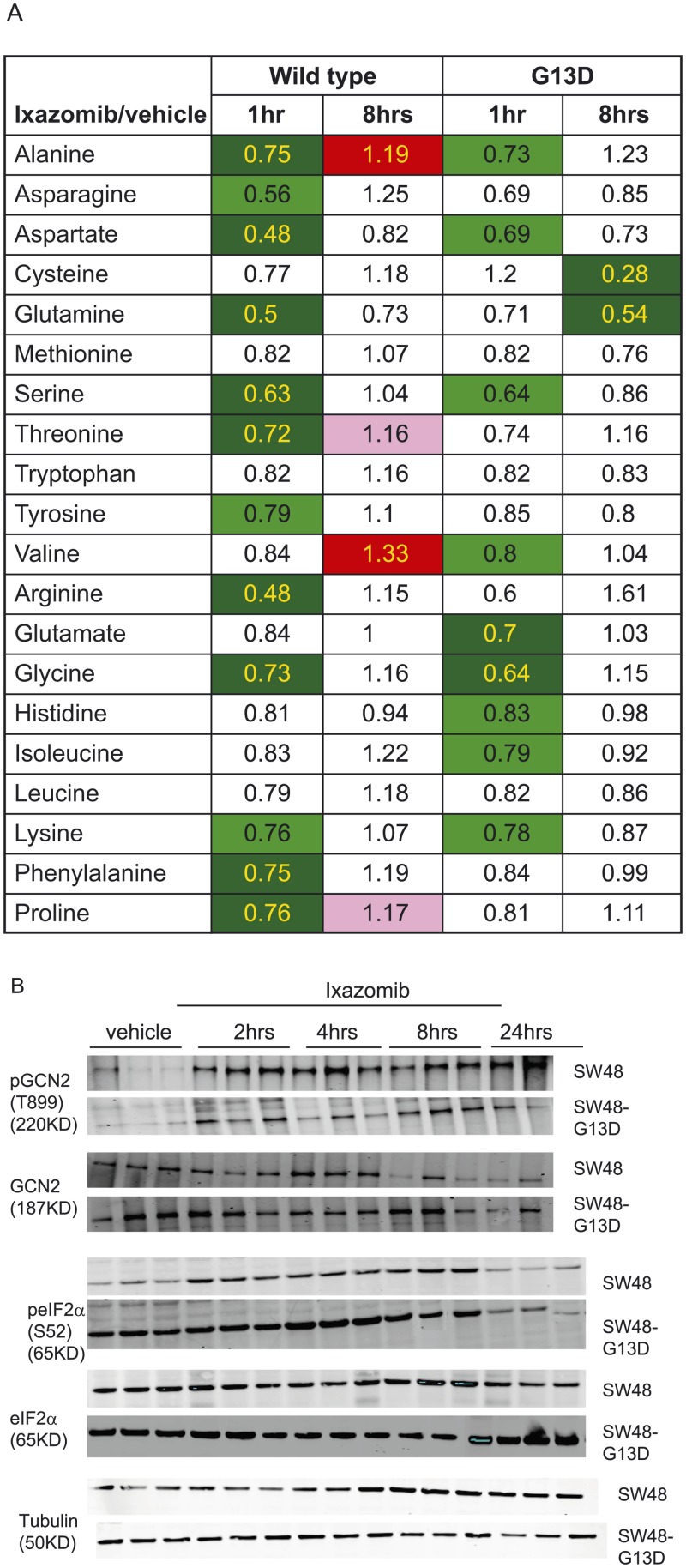
Ixazomib effect on amino acid metabolism. (A) Relative level of amino acids at 1 hr and 8hrs following ixazomib treatment of SW48 KRAS WT and G13D mutant tumors. The numbers are expressed as relative level of each amino acid following ixazomib treatment compared to vehicle treatment at that time point. Each data point is an average of tumors from five different animals. Color coding is similar as described in Fig 4. (B) Expression of pGCN2 (T899), GCN2, peIF2α (Ser52) and eIF2α in tumors from vehicle or ixazomib treatment. The vehicle samples were collected at 4hrs after dosing and the ixazomib treated samples were collected at different time points (as indicated in the figure) after the drug treatment. Tubulin was used as a loading control. Note: for pGCN2/GCN2 western blot, only 2 samples were evaluated at 24hr timepoint and 3 samples were evaluated for all other markers and time points in this figure (14 samples used in GCN2/pGCN2 blots and 15 samples in the other blots).

There were a number of metabolic alterations observed following ixazomib treatment in both SW48 and SW48-G13D tumors. One of the major alterations was the acute drop in levels of many individual amino acids following ixazomib treatment (Fig 5). Fig 5A shows the regulation of 20 amino acids at 1 and 8 hrs following ixazomib treatment of KRAS WT and mutant SW48 tumors. A number of essential and non-essential amino acids decreased 1 hr after ixazomib treatment in both KRAS WT and mutant tumors. The amino acid levels returned close to baseline by 8 hrs, suggesting acute amino acid depletion following ixazomib treatment. Proteasomal degradation of proteins creates short peptides which can be enzymatically converted to free amino acids; blocking proteasome activity with ixazomib should therefore lead to a reduced pool of peptides and free amino acids.

Amino acid depletion causes an excess of free tRNA which can bind to GCN2 and lead to its autophosphorylation and activation [24]. When activated, GCN2 phosphorylates eIF2αat Serine 52 which inhibits eIF2α and thereby represses protein translation [25,26]. To track the consequences of amino acid depletion, we evaluated the GCN2/phospho-eIF2α axis by western blot in SW48 WT and SW48-G13D xenograft tumors following ixazomib treatment. As shown in Fig 5B, ixazomib treatment results in phosphorylation of GCN2 at T899 in both KRAS WT and mutant tumors in a time-dependent manner, with slightly higher levels of pGCN2 detected after treatment in SW48 WT tumors compared to their KRAS mutant counterparts. Levels of total GCN2 were similar in both SW48 and SW48-G13D tumors. Consistent with the phosphorylation and activation of GCN2, we observed an increase in phospho-eIF2α (Ser 52) in the same time frame in both SW48 and SW48-G13D tumors, although the baseline level of p-eIF2α was higher in SW48-G13D tumors compared to SW48 tumors. Additionally, the total level of eIF2α was slightly higher in SW48-G13D tumors compared to SW48 tumors, but did not change in either tumor following ixazomib treatment. Together, the metabolic profiling and western blot data suggest ixazomib treatment results in an acute drop in free amino acid pools, which activates GCN2 and thus results in the phosphorylation and inactivation of eIF2α, which could lead to decreased global protein translation. Because these changes occur in both KRAS WT and mutant tumors, they do not explain the difference in ixazomib sensitivity between KRAS WT and mutant tumors.

### Increased fatty acid beta oxidation in KRAS WT tumors following ixazomib treatment

**Fig 6:**
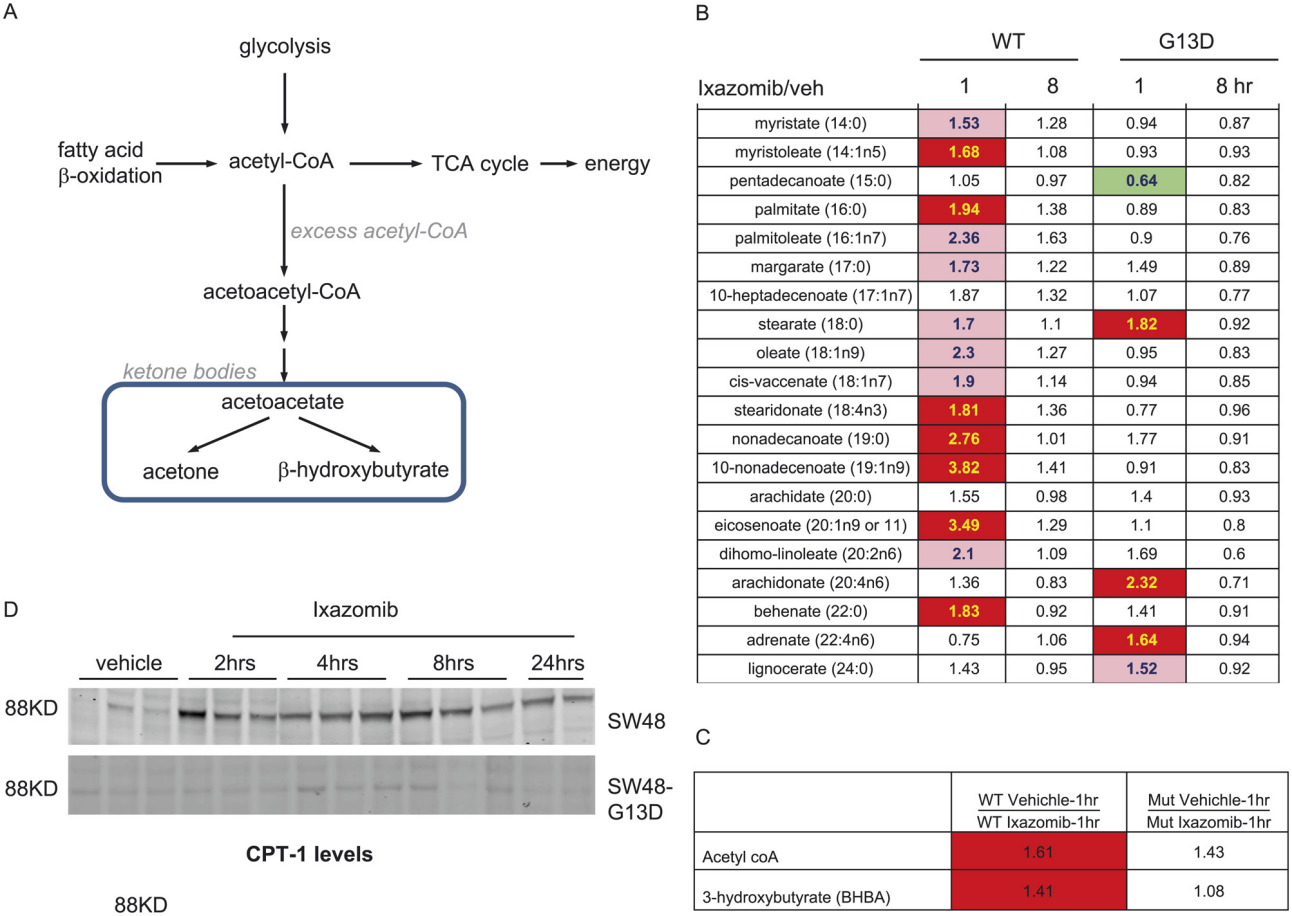
Induction of β-oxidation by ixazomib treatment in SW48 WT tumors. metabolites involved in fatty acid beta oxidation pathway. (A) Table summarizing free fatty acids in tumor samples. The numbers indicate the fold change in drug treated tumors over vehicle treated tumors at 1 and 8hrs time points. Each data point represents an average of tumors from five different animals. Table summarizing the levels of acetyl Co-A and 3 Hydroxy butyrate at 1hr after ixazomib treatment. The numbers indicate fold change in ixazomib treated tumors over vehicle treated tumors, n = 5 per data point. Changes in CPT-1 protein level following ixazomib treatment in SW48 and SW48-G13D tumors. The vehicle samples were collected at 4hrs after dosing and the ixazomib treated samples were collected at different time points (as indicated in the figure) after the drug treatment. The color coding is as described in Fig 4.

Utilization of fatty acids as an alternate energy source occurs when other catabolic pathways such as amino acid metabolism are compromised [27]. Free fatty acids can undergo β-oxidation to produce acetyl-coA and ketone bodies, as described in Fig 6A. In SW48 WT tumors, we detected increased levels of free fatty acids, acetyl-CoA, and the ketone body 3-hydroxybutyrate (3-BHBA) at 1 hr after ixazomib treatment (Fig 6B and 6C). The level of these metabolites returned close to baseline levels by 8 hrs of drug treatment. The increase in these biochemicals in KRAS WT tumors is reflective of a rapid shift to fatty acid β-oxidation following ixazomib treatment. In contrast, KRAS mutant tumors did not show signs of increased β-oxidation following PI treatment, with most free fatty acids, acetyl-CoA and 3-HBA levels remaining unchanged after ixazomib treatment.

To further evaluate the β-oxidation pathway in SW48 WT and KRAS mutant xenografts before and after ixazomib treatment, we determined the level of carnitine palmitoyl transferase 1 (CPT-1) by western blot (Fig 6D). CPT-1 is a mitochondrial membrane-bound enzyme that links carnitine to free fatty acids. The resulting fatty acyl carnitines then undergo β-oxidation in the mitochondria. Baseline levels of CPT-1 were very low in both in KRAS WT and mutant SW48 tumors. However, CPT-1 levels increased significantly with ixazomib treatment in KRAS WT tumors, but not in mutant tumors. This supports an enhanced capacity for β-oxidation in ixazomib-treated KRAS WT tumors.

### Regulation of lipid synthesis pathways by ixazomib treatment

**Fig 7:**
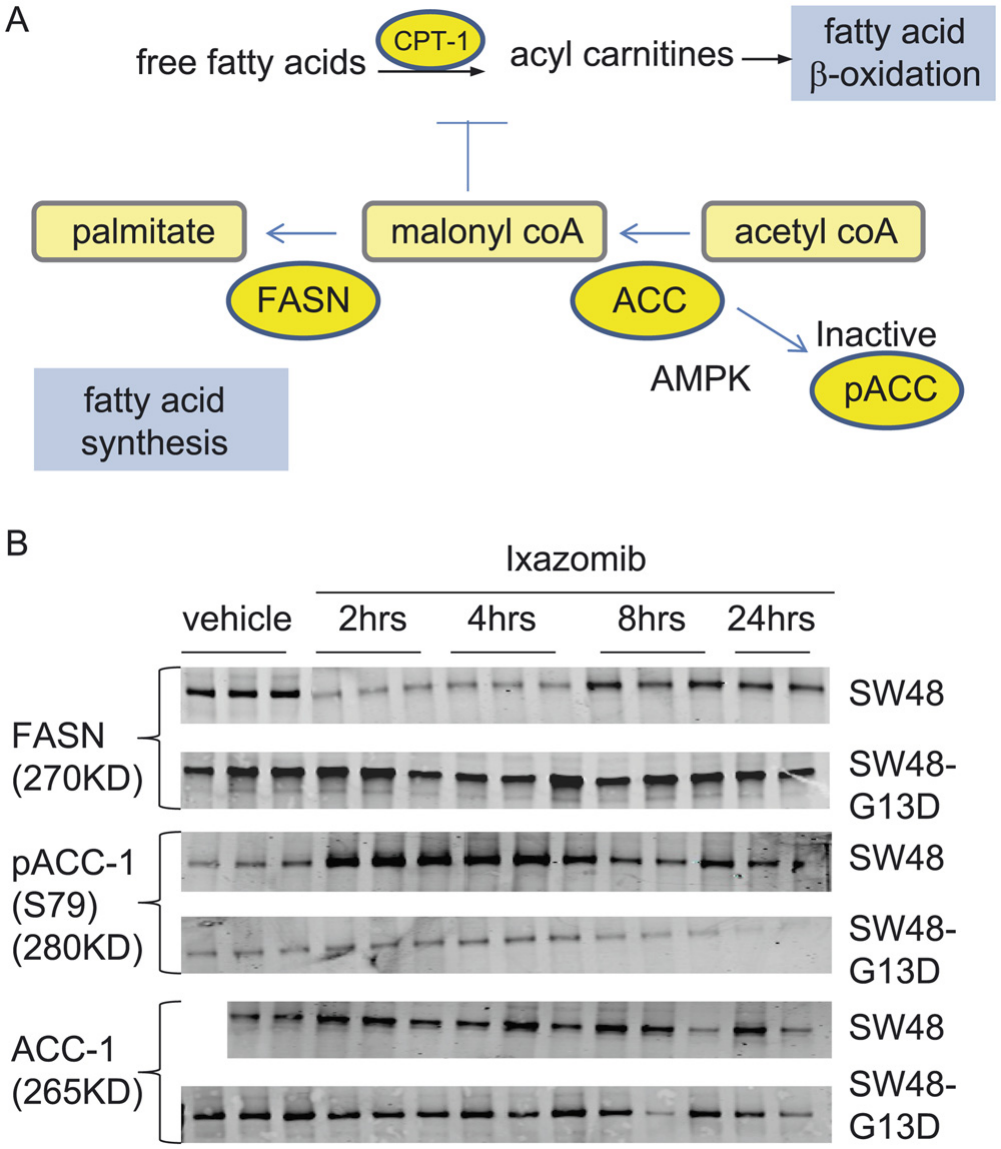
Ixazomib effect on key enzymes of lipid metabolism. (A) Enzymes and metabolites in lipid synthesis and β-oxidation pathways. (B) Regulation of FASN, pACC-1(S79) and ACC-1 proteins following vehicle and ixazomib treatment. The vehicle samples were collected at 4hrs after dosing and the ixazomib treated samples were collected at different time points (as indicated in the figure) after the drug treatment. (Note: For SW48 ACC1 blot there were two samples in vehicle group and thus there were only 14 samples for that blot)

To gain a more comprehensive understanding of lipid metabolism in the SW48 WT and KRAS mutant xenograft tumors at baseline and after ixazomib treatment, we evaluated the levels of enzymes involved in fatty acid production (Fig 7A), including fatty acid synthase (FASN). This enzyme complex converts malonyl CoA to palmitate, which then undergoes elongation and desaturation resulting in long chain and unsaturated fatty acids. There is a higher level of fatty acid synthase (FASN) protein at baseline in SW48-G13D tumors compared to SW48 WT tumors (Fig 7B). This is consistent with our metabolic profiling data, showing an elevated baseline level of long chain fatty acids in KRAS mutant tumors compared to KRAS WT tumors (Fig 4C). In KRAS WT tumors, ixazomib treatment resulted in decreased levels of FASN between 2–8 hrs of treatment, which would likely decrease lipogenesis in these tumors. Interestingly, the level of FASN did not drop so significantly in SW48-G13D tumors after ixazomib treatment, suggesting that fatty acid synthesis would be maintained in the SW48-G13D xenografts.

Malonyl CoA has opposing roles in fatty acid synthesis and beta oxidation (Fig 7A). In fatty acid synthesis, as described above, malonyl CoA is a key building block of fatty acids. In contrast, malonyl CoA inhibits β-oxidation by inhibiting CPT-1. Levels of malonyl CoA are regulated by acetyl CoA carboxylase [19], which converts acetyl CoA to malonyl CoA. There are two isoforms of ACC. While ACC-1 is ubiquitously expressed in different organs, expression of ACC-2 is limited. Our mRNA and western blot data showed only ACC-1 expression in SW48 isogenic tumors (data not shown). We therefore evaluated the level and phosphorylation status of ACC-1 in the xenograft tumors treated with ixazomib.

The unphosphorylated form of ACC-1 is the active form, while its phosphorylation at ser 79 (primarily by AMPK) deactivates the enzyme [28]. In SW48 WT tumors, the level of pACC-1 increased several fold following ixazomib treatment, (Fig 7B) but remained unchanged or even decreased in SW48 KRAS mutant tumors. These data suggest that ACC-1 remains active and unphosphorylated in SW48 KRAS mutant tumors, providing a source of malonyl CoA for fatty acid synthesis. In contrast, the increase in pACC-1 (the inactive form) in the KRAS WT tumors would decrease the level of malonyl CoA available for fatty acid synthesis, and at the same time relieve the malonyl CoA-dependent inhibition of CPT-1, thereby promoting β-oxidation.

We also evaluated protein levels of some of these key enzymes in the KRAS WT and mutant primary human NSCLC xenografts, PHTX132Lu and PHTX192Lu. Higher baseline levels of FASN were observed in KRAS-G13D mutant PHTX192Lu than in KRAS WT PHTX132Lu. After ixazomib treatment, PHT132Lu tumors demonstrated an increase in pACC-1 and CPT-1, whereas these proteins remained at low levels in the PHTX192Lu tumors (S2 Fig). The regulation of fatty acid metabolism enzymes by ixazomib in these primary human lung xenografts therefore follows the same pattern as in the SW48 isogenic tumor pairs.

## Discussion

In this study, we discovered a correlation between *KRAS* genotype and *in vivo* response to the proteasome inhibitor ixazomib in a heterogeneous group of 14 NSCLC and 6 colon xenograft models. Ixazomib demonstrated more antitumor activity in tumors with WT KRAS than in tumors with mutant KRAS. Using an isogenic system, we confirmed that the introduction of a KRAS G13D or G12V mutation in the KRAS WT SW48 colon cancer cell line conferred resistance to ixazomib *in vivo*. The similar ixazomib concentrations and 20S proteasome inhibition in SW48 WT and G13D isogenic tumors suggests that KRAS pathway activation does not alter tumor exposure or target inhibition by ixazomib, but rather affects cellular response to proteasome inhibitor-induced stress.

We chose to evaluate this hypothesis in an *in vivo* system, because the association between KRAS mutation and resistance to PI was not observed *in vitro* in our experiments with the isogenic SW48 cell lines, or in a group of 20 (17 primary and 3 cell line derived) NSCLC models evaluated using soft agar colony formation assays. In contrast to our results, a previous *in vitro* study in DLD1 and HCT116 KRAS mutant and WT isogenic cell lines [29] reported the opposite association: in that study, proteasome inhibition by small molecules (bortezomib and MG132) or shRNAs against proteasome subunits had a greater effect on the viability of KRAS mutant cells than on the KRAS WT cells. The reason for this difference is unknown, but there may be a difference between introducing mutant KRAS into a WT background, as was done in SW48 isogenic lines, vs. deleting the mutant copy of KRAS, leaving a cell line with a WT/- genotype.

Because the *in vitro* and *in vivo* systems showed striking differences in the effect of KRAS mutation on PI sensitivity, we explored metabolic differences, a facet of RAS signaling which would be heavily influenced by the tumor environment. Previous work has identified many aspects of metabolism affected by RAS, including promotion of glucose uptake [4,15,16,22,30], glycolytic flux, and channeling of intermediates into the PPP and hexosamine biosynthesis pathways [4]. Some of these changes are detectable at the level of gene expression, as shown in studies where RAS activation promotes expression of a number of metabolic genes, including GLUT1 and also those in pathways for sterol biosynthesis, pyrimidine metabolism and O-glycan biosynthesis [4,15,29]. Here, we evaluated the levels of a broad array of metabolites present in SW48 and SW48 KRAS G13D xenograft tumors. To further characterize metabolic pathways, we also investigated levels of key proteins in lipid metabolism.

The baseline comparison between KRAS WT and mutant tumors showed differences consistent with published *in vitro* work. We detected reduced levels of glucose and glycogen intermediates in KRAS mutant tumors, which is suggestive of increased glucose metabolism in KRAS mutant tumors. Higher levels of GLUT4 in KRAS mutant tumors suggest one mechanism for this effect. Other key differences observed between KRAS WT and mutant tumors at baseline were the levels of free fatty acids and of a pivotal enzyme in the lipogenesis pathway, FASN. Proliferating cells need long chain fatty acids and phospholipids for membrane biogenesis, and thus enhanced fatty acid synthesis is a hallmark of many type of cancers [31]. In many tumor types, elevated FASN expression is associated with tumor progression and poor prognosis [32,33]. At baseline, KRAS mutant SW48 tumors showed metabolic features that indicate a more aggressive phenotype, elevated redox potential and drug resistance. Increased glucose metabolism, lipid synthesis, and glutathione synthesis were observed in mutant tumors, all of which could potentially influence the sensitivity to PI by enabling continued proliferation under proteotoxic stress or better adaptation to stresses induced by proteasome inhibition.

In the current study we report metabolic changes caused by PI treatment and also how these effects differ between KRAS WT and mutant tumors. Similar to the observation of Suraweera et al, [34] where bortezomib treatment of NIH3T3 cells *in vitro* resulted in a drop in amino acid levels and activation of GCN2-mediated signaling, we detected a drop in AA levels, activation of GCN2 and phosphorylation of eIF2α following ixazomib treatment in both KRAS mutant and WT SW48 tumors, which would result in inhibition of global protein translation. Our data also show regulation of lipid metabolism by proteasome inhibitor treatment *in vivo* and differential effects on lipid metabolism between KRAS WT and mutant tumors. Notably, in KRAS WT tumors FASN expression decreased with ixazomib treatment, while in KRAS mutant tumors FASN remained high. This could provide a growth advantage to KRAS mutant tumors under conditions of PI treatment, as FASN is reported to induce a number of tumorigenic pathways and inhibit apoptosis[32,33]. The mechanism of decreased FASN in KRAS WT tumors after ixazomib treatment is not yet known, but bortezomib has been reported to reduce mRNA expression of the transcription factor SREBP1c and the expression of its target genes such as FASN, ACC, and HMGCoA synthase in a rat model of alcohol induced fatty liver [35]. Although we also found a decrease in SREPB1 protein level (data not shown), we did not detect changes in either FASN or SREBP at the mRNA level (data not shown). Additional experiments are needed to understand the mechanism of decreased FASN protein.

Another difference observed between KRAS WT and mutant tumor response to PI involved the lipid β-oxidation pathway. Through β-oxidation, lipids can be utilized as an energy source, but compared to glycolysis and oxidative phosphorylation, β-oxidation is an inefficient means of energy production and is not usually used by cancer cells. Acetyl coA produced by β-oxidation is used to produce ketone bodies, and thus the level of ketone bodies reflects the level of cellular β-oxidation. Ketone bodies usually cannot be consumed by tumor cells [36], although highly aggressive and metastatic tumors can utilize ketone bodies as an energy source [37]. Elevated levels of ketone bodies and elevated protein levels of two key regulators of β-oxidation, pACC1 (Ser59) and CPT-1, were detected in KRAS WT tumors after ixazomib treatment. ACC-1 converts acetyl coA to malonyl coA, and the latter inhibits CPT-1 function. Inactivating phosphorylation of ACC-1 would relieve the CPT-1 inhibition, thus promoting β-oxidation. The data suggest that KRAS WT tumors use β-oxidation as a source of energy under ixazomib treatment. This apparent attempt to compensate for the loss of more efficient sources of energy does not ultimately seem to help KRAS WT tumors to survive the stress. In contrast, KRAS mutant tumors did not activate the less-efficient β-oxidation process, but rather continued lipid synthesis supporting cell growth and survival.

In conclusion, the data presented here has implications for future investigation of PIs in solid tumors, especially in NSCLC and colon cancer. There may be utility for a patient enrichment strategy based on *KRAS* genotype. However, our preclinical data in NSCLC and colon models supporting this approach may not apply broadly to other solid tumor types, or in hematological cancers. Recent clinical data [19] in multiple myeloma patients presents an interesting case, where *NRAS* but not *KRAS* mutations were associated with lower response to single-agent bortezomib. This may be due to either functional differences between KRAS and NRAS as oncogenic drivers or the different biology of myeloma compared to solid tumors. Interestingly, KRAS mutant MM xenograft models (MM.1S and RPMI-8226) are highly sensitive to ixazomib [38],[39], suggesting that the PI-resistance of KRAS mutant tumors is indication specific. Future studies will be needed to compare the metabolic response between KRAS- and NRAS-driven solid tumor and MM models to more broadly assess the role of RAS mutations in proteasome inhibitor response.

## Supporting Information

S1 Fig ATF3 regulation with ixazomib. ATF3 protein regulation by IHC at different time points after ixazomib treatment in KRAS WT and KRAS mutant tumor. Each bar represents the fold change in ixazomib treated tumors compared to vehicle treated tumors and average data from three different tumors +/- SD.
(DOCX)

S2 Fig Western blot analysis of lipid pathway markers in PHTX132Lu and PHTX192Lu tumors. Expression of FASN, pACC-1(S79), ACC-1 and CPT-1 in tumor extracts from PHTX132Lu and PHTX192Lu tumors. The vehicle samples were collected at 4hrs after treatment and the ixazomib treated samples were collected at different time points (as indicated in the figure) after the drug treatment.
(DOCX)

S1 Table Details of each xenograft models used in this manuscript. (DOCX)

S2 Table Genes, and number of mutational assays per gene, included in the OncoCarta^™^ custom panel. Genes included in custom Oncocarta panel which was used for mutation analysis by Sequenom. Genes with * were included in Oncocarta V1. The numbers in the parentheses indicate the number of distinct assays for mutation detection per gene.
(DOCX)

S3 Table Different genetic mutations detected in xenograft tumors. (DOCX)

S4 Table Activity of ixazomib in colony formation assay (CFA). RAS genotype and activity of ixazomib in 20 (17 primary and 3 cell line derived) NSCLC tumor explants determined by CFA assay. IC_50_ is the concentration in nM for 50% inhibition in colony formation
(DOCX)
