# Supplementary material for: KRAS Genotype Correlates with Proteasome Inhibitor Ixazomib Activity in Preclinical In Vivo Models of Colon and Non-Small Cell Lung Cancer: Potential Role of Tumor Metabolism
Source: PLoS One. 2015 Dec 28;10(12):e0144825. doi: 10.1371/journal.pone.0144825 (PMC4692403; doi:10.1371/journal.pone.0144825)

**S1 Fig. ATF3 regulation with ixazomib.** ATF3 protein regulation by IHC at different time points after ixazomib treatment in KRAS WT and KRAS mutant tumor. Each bar represents the fold change in ixazomib treated tumors compared to vehicle treated tumors and average


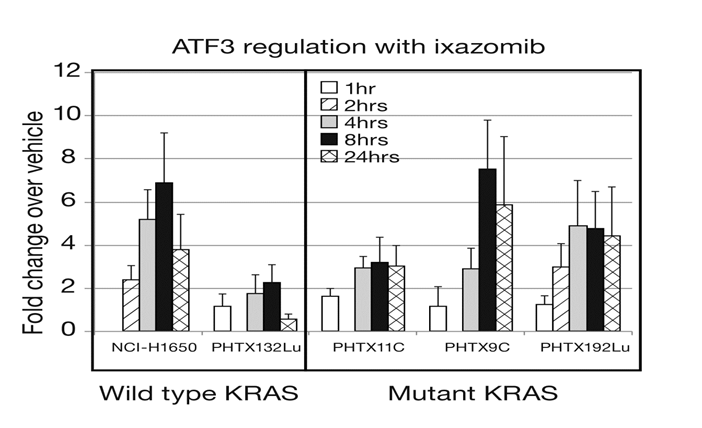

Supplement: S1 Fig — ATF3 protein regulation by IHC at different time points after ixazomib treatment in KRAS WT and KRAS mutant tumor. Each bar represents the fold change in ixazomib treated tumors compared to vehicle treated tumors and average data from three different tumors +/- SD. (DOCX) [file pone.0144825.s001.docx]
