# Supplementary material for: KRAS Genotype Correlates with Proteasome Inhibitor Ixazomib Activity in Preclinical In Vivo Models of Colon and Non-Small Cell Lung Cancer: Potential Role of Tumor Metabolism
Source: PLoS One. 2015 Dec 28;10(12):e0144825. doi: 10.1371/journal.pone.0144825 (PMC4692403; doi:10.1371/journal.pone.0144825)

**S2 Fig. Western blot analysis of lipid pathway markers in PHTX132Lu and PHTX192Lu tumors.** Western blot analysis of lipid pathway markers in PHTX132Lu and PHTX192Lu tumors. Expression of FASN, pACC-1(S79), ACC-1 and CPT-1 in tumor extracts from PHTX132Lu and PHTX192Lu tumors. The vehicle samples were collected at 4hrs after treatment and the ixazomib treated samples were collected at different time points (as indicated in the figure) after the drug treatment.


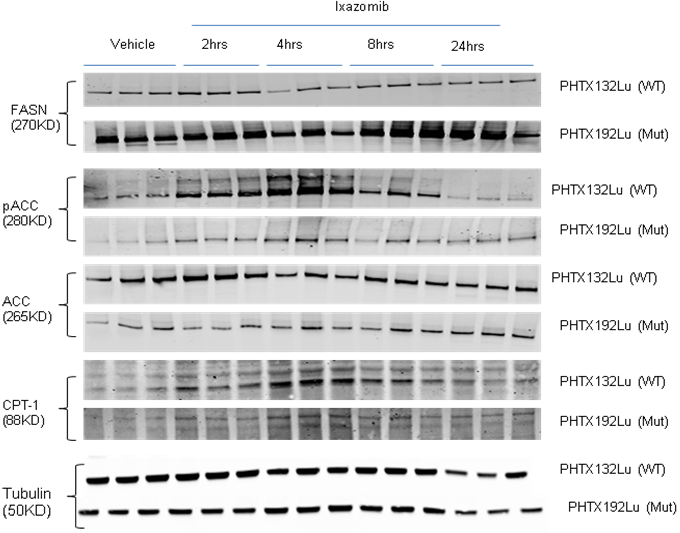

Supplement: S2 Fig — Expression of FASN, pACC-1(S79), ACC-1 and CPT-1 in tumor extracts from PHTX132Lu and PHTX192Lu tumors. The vehicle samples were collected at 4hrs after treatment and the ixazomib treated samples were collected at different time points (as indicated in the figure) after the drug treatment. (DOCX) [file pone.0144825.s002.docx]
