# Supplementary material for: KRAS Genotype Correlates with Proteasome Inhibitor Ixazomib Activity in Preclinical In Vivo Models of Colon and Non-Small Cell Lung Cancer: Potential Role of Tumor Metabolism
Source: PLoS One. 2015 Dec 28;10(12):e0144825. doi: 10.1371/journal.pone.0144825 (PMC4692403; doi:10.1371/journal.pone.0144825)
Supplement: S1 Table — (DOCX) [file pone.0144825.s003.docx]

**S1 Table. Details of each xenograft models used in this manuscript**

| **Xenograft model** | **Indication** | **Cell line/primary** | **Mice strain** | **Number of cells** | **Matrigel (1:1)** | **MTD (IV, BIW) mg/kg** |
| --- | --- | --- | --- | --- | --- | --- |
| LXFE409 | NSCLC | Primary | NMRI nu/nu | NA | NA | 13 |
| HCC827 | NSCLC | Cell line | NCr-Nude | 6 X 10^6^ | Yes | 13 |
| PHTX132Lu | NSCLC | Primary | CB17-SCID | NA | NA | 14 |
| NCI-H1975 | NSCLC | Cell line | NCr-Nude | 1 x 10^6^ | Yes | 13 |
| LXFA677 | NSCLC | Primary | NMRI nu/nu | NA | NA | 13 |
| PHTX24C | Colon | Primary | NCr-Nude | NA | NA | 14 |
| PHTX21C | Colon | Primary | CB17-SCID | NA | NA | 11 |
| H1650 | NSCLC | Cell line | Balb/c nu/nu | 2 x 10^6^ | Yes | 11 |
| PHTX9C | Colon | Primary | CB17-SCID | NA | NA | 13 |
| LXFL1121 | NSCLC | Primary | NMRI nu/nu | NA | NA | 13 |
| LXFL1674 | NSCLC | Primary | NMRI nu/nu | NA | NA | 13 |
| LXFA1041 | NSCLC | Primary | NMRI nu/nu | NA | NA | 13 |
| PHTX11C | Colon | Primary | CB17-SCID | NA | NA | 13 |
| PHTX192Lu | NSCLC | Primary | CB17-SCID | NA | NA | 14 |
| Calu-6 | NSCLC | Cell line | NCr-Nude | 5 X 10^6^ | No | 14 |
| A549 | NSCLC | Cell line | Balb/c nu/nu | 5 X 10^6^ | Yes | 11 |
| NCI-H358 | NSCLC | Cell line | Balb/c nu/nu | 5 X 10^6^ | Yes | 11 |
| HCT116 | Colon | Cell line | NCr-Nude | 2 X 10^6^ | No | 14 |
| NCI-H460 | NSCLC | Cell line | NCr-Nude | 2.5 X 10^6^ | No | 14 |
| PHTX17C | Colon | Primary | NCr-Nude | NA | NA | 14 |
| SW48 | Colon | Cell line | NCr-Nude | 2 x 10^6^ | Yes | 13 |
| SW48-KRAS-G13D | Colon | Cell line | NCr-Nude | 2 x 10^6^ | Yes | 13 |
