# Supplementary material for: KRAS Genotype Correlates with Proteasome Inhibitor Ixazomib Activity in Preclinical In Vivo Models of Colon and Non-Small Cell Lung Cancer: Potential Role of Tumor Metabolism
Source: PLoS One. 2015 Dec 28;10(12):e0144825. doi: 10.1371/journal.pone.0144825 (PMC4692403; doi:10.1371/journal.pone.0144825)
Supplement: S2 Table — Genes included in custom Oncocarta panel which was used for mutation analysis by Sequenom. Genes with * were included in Oncocarta V1. The numbers in the parentheses indicate the number of distinct assays for mutation detection per gene. (DOCX) [file pone.0144825.s004.docx]

**S2 Table. Genes, and number of mutational assays per gene, included in the OncoCarta™ custom panel.** Genes included in custom Oncocarta panel which was used for mutation analysis by Sequenom. Genes with * were included in Oncocarta V1. The numbers in the parentheses indicate the number of distinct assays for mutation detection per gene.

| **Gene** | **Number of mutational assays per gene** |
| --- | --- |
| ABL1^a^ | 16 |
| AKT1 ^a^ | 9 |
| AKT2 ^a^ | 2 |
| APC | 12 |
| BRAF^a^ | 44 |
| CDKN2A | 7 |
| CDK ^a^ | 2 |
| CSFR1 | 4 |
| CTNNB1 | 27 |
| EGFR^a^ | 74 |
| ERBB2 ^a^ | 8 |
| ERBB3 | 1 |
| FBX4 | 6 |
| FBXW7 | 4 |
| FGFR1 ^a^ | 2 |
| FGFR2 | 2 |
| FGFR3^a^ | 6 |
| FLT3 ^a^ | 7 |
| GNAQ | 1 |
| HRAS ^a^ | 6 |
| JAK2^a^ | 1 |
| JAK3 | 3 |
| KIT^a^ | 69 |
| KRAS^a^ | 16 |
| MAP2K1 | 5 |
| MAP2K2 | 5 |
| MET^a^ | 11 |
| MLH1 | 1 |
| MYC | 6 |
| NRAS^a^ | 10 |
| PDGFRA^a^ | 27 |
| PIK3CA^a^ | 39 |
| PTEN | 12 |
| PTPN11 | 1 |
| RB1 | 11 |
| RET ^a^ | 20 |
| SOS1 | 3 |
| SRC | 1 |
| STK11 | 11 |
| TP53 | 15 |
| VHL | 7 |

^a^genes included in OncoCarta™ V1
