# Supplementary material for: KRAS Genotype Correlates with Proteasome Inhibitor Ixazomib Activity in Preclinical In Vivo Models of Colon and Non-Small Cell Lung Cancer: Potential Role of Tumor Metabolism
Source: PLoS One. 2015 Dec 28;10(12):e0144825. doi: 10.1371/journal.pone.0144825 (PMC4692403; doi:10.1371/journal.pone.0144825)
Supplement: S3 Table — (DOCX) [file pone.0144825.s005.docx]

**S3 Table. Different genetic mutations detected in xenograft tumors**

| **Xenograft model** | **T/C^a^** | **Ras status** | **Other mutations** |
| --- | --- | --- | --- |
| LXFE409^C^ | 0.07 | WT | None detected |
| HCC827 | 0.26 | WT | EGFR_E746_A750DEL^b^; EGFR_T751A^b^ |
| PHTX132Lu | 0.29 | WT | VHL_P81S |
| NCI-H1975 | 0.4 | WT | EGFR_L858R^b^; EGFR_T790M^b^;TP53_R248W;TP53_R273H |
| LXFA677^C^ | 0.42 | WT | None detected |
| PHTX24C | 0.43 | Kras A146T | None detected |
| PHTX21C | 0.48 | WT | BRAF_V600E^b^; PIK3CA_E545K^b^ |
| NCI-H1650 | 0.55 | WT | EGFR_E746_T751DEL^b^ |
| PHTX9C | 0.64 | Kras G12D | None detected |
| LXFL1121^C^ | 0.68 | WT | None detected |
| LXFL1674^C^ | 0.69 | Kras G12C | None detected |
| LXFA1041^C^ | 0.75 | Kras G12V | None detected |
| PHTX11C | 0.77 | Kras Q61H | APC_E1309FS; TP53_R273C |
| PHTX-192Lu | 0.8 | Kras G13D | STK11_D194N |
| Calu-6 | 0.84 | Kras Q61K | None detected |
| A549 | 0.9 | Kras G12S | STK11_Q37 |
| NCI-H358 | 0.92 | Kras G12C | None detected |
| HCT116 | 0.94 | Kras G13D | PIK3CA_H1047R^b^; CTNNB1_S45P |
| NCI-H460 | 1.1 | Kras Q61H | PIK3CA_E545K^b^; STK11_Q37 |
| PHTX17C | 1.1 | Kras G12V | None detected |

^a^average volume of treated tumors/average volume of control tumors

^b^genes included in OncoCarta™ V1

^C^ models tested with OncoCarta^TM^ V1
