# Supplementary material for: KRAS Genotype Correlates with Proteasome Inhibitor Ixazomib Activity in Preclinical In Vivo Models of Colon and Non-Small Cell Lung Cancer: Potential Role of Tumor Metabolism
Source: PLoS One. 2015 Dec 28;10(12):e0144825. doi: 10.1371/journal.pone.0144825 (PMC4692403; doi:10.1371/journal.pone.0144825)
Supplement: S4 Table — RAS genotype and activity of ixazomib in 20 (17 primary and 3 cell line derived) NSCLC tumor explants determined by CFA assay. IC50 is the concentration in nM for 50% inhibition in colony formation (DOCX) [file pone.0144825.s006.docx]

**S4 Table. Activity of ixazomib in colony formation assay (CFA).** RAS genotype and activity of ixazomib in 20 (17 primary and 3 cell line derived) NSCLC tumor explants determined by CFA assay. IC_50_ is the concentration in nM for 50% inhibition in colony formation

data from three different tumors +/- SD.

| **Tumor** | **RAS mutation** | **IC_50_ (nM)** | **Tested as xenograft** |
| --- | --- | --- | --- |
| LXFA623 | None | 34 |  |
| LXFACALU6LX | KRAS Q61K | 38 | Y |
| LXFL1674 | KRAS G12C | 38 | Y |
| LXFA1041 | KRAS G12V | 39 | Y |
| LXFA586 | None | 50 |  |
| LXFL529 | NRAS Q61H | 60 |  |
| LXFE690 | None | 61 |  |
| LXFAA549LX | KRAS G12S | 70 | Y |
| LXFLH460LX | KRAS Q61H | 76 | Y |
| LXFL430 | None | 85 |  |
| LXFL1121 | None | 92 | Y |
| LXFA297 | None | 99 |  |
| LXFA400 | None | 100 |  |
| LXFA983 | KRAS G12C | 102 |  |
| LXFE409 | None | 110 | Y |
| LXFA677 | None | 115 | Y |
| LXFE397 | None | 148 |  |
| LXFA1335 | KRAS G12C | 166 |  |
| LXFL1176 | KRAS G12V | 175 |  |
| LXFA289 | None | 272 |  |
